# Gold–Carbon Nanocomposites for Environmental Contaminant Sensing

**DOI:** 10.3390/mi12060719

**Published:** 2021-06-19

**Authors:** Shahrooz Rahmati, William Doherty, Arman Amani Babadi, Muhamad Syamim Akmal Che Mansor, Nurhidayatullaili Muhd Julkapli, Volker Hessel, Kostya (Ken) Ostrikov

**Affiliations:** 1 School of Chemistry and Physics, Queensland University of Technology (QUT), Brisbane 4000, Australia; kostya.ostrikov@qut.edu.au; 2 Centre for Agriculture and the Bioeconomy, Institute for Future Environments, Queensland University of Technology (QUT), Brisbane 4000, Australia; w.doherty@qut.edu.au; 3 Centre for Material Science, Queensland University of Technology (QUT), Queensland, Brisbane, Brisbane 4000, Australia; 4 Nanotechnology & Catalysis Research Centre (NANOCAT), Institute of Graduate Studies, University of Malaya, Kuala Lumpur 50603, Malaysia; syamim.akmal93@gmail.com; 5 Functional Omics and Bioprocess Development Laboratory, Institute of Biological Sciences, Faculty of Science, University of Malaya, Kuala Lumpur 50603, Malaysia; arman_amani@um.edu.my; 6 School of Chemical Engineering and Advanced Materials, The University of Adelaide, Adelaide 5005, Australia; volker.hessel@adelaide.edu.au; 7 School of Engineering, University of Warwick, Library Rd, Coventry CV4 7AL, UK

**Keywords:** gold–carbon nanocomposites, environmental monitoring, sensing, pollutant

## Abstract

The environmental crisis, due to the rapid growth of the world population and globalisation, is a serious concern of this century. Nanoscience and nanotechnology play an important role in addressing a wide range of environmental issues with innovative and successful solutions. Identification and control of emerging chemical contaminants have received substantial interest in recent years. As a result, there is a need for reliable and rapid analytical tools capable of performing sample analysis with high sensitivity, broad selectivity, desired stability, and minimal sample handling for the detection, degradation, and removal of hazardous contaminants. In this review, various gold–carbon nanocomposites-based sensors/biosensors that have been developed thus far are explored. The electrochemical platforms, synthesis, diverse applications, and effective monitoring of environmental pollutants are investigated comparatively.

## 1. Introduction

During recent years, the evolution of nanomaterials-based analytical methods has made remarkable advances for various important applications such as fundamental biological analysis [1,2], medical and clinical diagnostics [3,4,5], pharmaceutical analysis [6,7,8], monitoring of health [9,10,11], food safety [12,13,14], and environmental monitoring [15,16,17]. Monitoring the different hazardous pollutants in surrounding environmental elements (air, land, and water) is one of the most important aspects of public health and environmental care. It has generated considerable scientific interest and social concern to protect the environment from the impact of the distribution of natural/industrial organic and inorganic contaminants [18]. Nanoscience and nanotechnology are the most recent advanced scientific fields that can serve humankind in this purpose [19].

Nowadays, the emerging nanoscience and nanotechnology techniques have provided insight to design novel nanocomposite materials with unique properties and structures to achieve cooperatively enhanced performance to promote the detection, identification, and tracking of environmental matters [20]. A wide range of regulated and unregulated natural and chemical materials have posed a threat to environmental security, including inorganic gases (carbon monoxide (CO), carbon dioxide (CO_2_), sulphur dioxide (SO_2_), nitric oxide (NO), etc.), volatile and semi-volatile organic compounds (aldehydes, ketones, hydrocarbons), heavy metals (As(III), Zn(II), Cd(II), Cu(II), Ni(II), Pb(II), Hg(II), As(V), etc.) and persistent organic pollutants (POPs) (aldrin, chlordane, dieldrin, heptachlor (DDT), toxaphene, endrin, mirex, hexachlorobenzene, polychlorinated biphenyls (PCB), furans, dioxins, heptachlor, etc.) which are produced by human and animal faecal waste, industry and agriculture activities, natural toxins, etc. [21,22,23].

The most common analytical techniques for the quantification and identification of chemical pollutants are flame atomic absorption spectrometry (FAAS) [24,25], atomic absorption spectrometry (AAS) [26,27], gas/liquid chromatography–mass spectrometry [28,29], inductively coupled plasma mass spectroscopy (ICP–MS) [30,31], inductively coupled plasma atomic emission spectroscopy (ICP–AES) [32,33], high performance liquid chromatography (HPLC)–fluorescence (FL) [34,35] and ultraviolet (UV) detection [36,37], and quantitative polymerase chain reaction (qPCR) [38,39]. Although the above-mentioned analytical protocols have shown very high sensitivity and specificity, these techniques are limited by either a long procedure of sample preparation or complicated equipment. Therefore, we categorised them as time-consuming techniques. Moreover, these instruments are very expensive, and a high level of expertise is essential to perform an analytical analysis using each of them. In addition, online and real-time monitoring and sensing of different chemicals with outstanding sensitivity and spatial resolution are highly required [38]. The design and development of a low-cost, flexible, sensitive, and mobile monitoring system is a critical task. Nanomaterials with unique physicochemical properties have an incredible potential for designing detecting devices and providing a solution for pollutant elimination [21].

In recent years, growing attention has been paid to nanoscale materials research, with a particular focus on metallic nanoparticles and their applications in environmental analysis [40,41]. Metal nanoparticles (MNPs) have a high surface area ratio of atoms with free valences to the cluster of total atoms, which allows them to enhance and improve the activity of chemical reactions. Furthermore, size controllability, conductivity, magnetism, light-absorbing, mechanical strength, chemical stability, emitting properties, and surface tenability provide a perfect platform for developing such a nanostructure in sensing and catalytic applications [42]. MNPs are increasingly used in many electrochemical, electroanalytical, and bioelectronic applications owing to their extraordinary electrocatalytic properties [43]. Over the last decade, NPs electrochemical behaviour and applications have enhanced remarkably [42]. As a result, the fabrication of advanced sensitive electrochemical sensors and biosensors with the incorporation of nanostructured materials is important [44]. Among the most extensive research on metallic NPs, gold nanomaterials (AuNMs) have received considerable research attention in the electrochemical field due to their advantages in catalysis, mass transport, good interface-dominated properties, highly effective surface area, and control over the surrounding environment [45]. Additionally, the utilisation and prospects of AuNMs including gold nanoparticles (AuNPs), gold nanoclusters (AuNCs), gold nanoporous (AuNPG), and their various nanocomposites provide a great opportunity to increase AuNM applications further in electrochemical platforms for environmental monitoring [38].

Nanocomposites are combinations of different kinds of nanomaterials with other molecules or nanoscale materials, such as nanotubes or nanoparticles. Generally, these unique structures possess novel physicochemical properties which develop new types of applications [46,47]. During recent years, numerous gold-based nanocomposites have been established as follows: gold–carbon nanotube nanocomposites [48,49,50], polymer–gold nanocomposites [51,52,53], graphene–gold nanocomposites [54,55,56], biomolecule–gold nanocomposites [57,58], metal oxide–gold nanocomposites [59,60]. Different gold-based nanocomposites have been used in diverse fields such as sensors [61,62,63], biosensors [64,65,66], optics [67,68,69], and medical areas [70,71,72]. Hybridisation of MNPs and carbon-based materials is considered as a new approach for creating unique hybrid materials with novel properties for a variety of applications (e.g., gas sensors, catalysts, magnetic and electronic devices) [73]. Carbon-based nanomaterials (CBNs) such as graphenes (GRs), graphene oxide (GO), reduced graphene oxide (rGO), carbon nanotubes (CNTs), single-wall carbon nanotubes (SWCNTs), multi-wall carbon nanotubes (MWCNTs), ordered graphitised mesoporous carbons (GMCs), carbon nanofibers (CNFs), and carbon nanohorns (CNHs) have tremendous research interest and play an important role in the development of a variety of nanocomposites. Their remarkable properties, such as large surface area, excellent electrical conductivity, high chemical, and thermal stability, and strong mechanical strength, make CBNs an ideal candidate to be used as a support in gold nanocomposites matrix to fabricate various electrochemical biosensors for analysis of environmental contaminants [20]. 

A possible strategy for overcoming the inherent limitations of carbon and gold as sub-materials in environmental detection applications is to combine them in the form of nanocomposites. The synthesis of nanocomposites-based electrode materials comprising AuNPs and CBNs has provided enhanced sensitivity and limit of detection (LOD). In particular, CNT and GR materials have drawn special attention owing to their low electrical resistivity and high electron mobility, offering a strong enhancement to the electrical conductivity of electronic materials. They have been used as a sensing element in various classes of electrochemical sensors/biosensors to achieve a low LOD in a wide range of environmental applications [38]. This review focuses on the most recent applications of gold–carbon nanocomposites in the design and synthesis of various electrochemical platforms-based sensors and biosensors for the detection, identification, and quantification of emerging chemical contaminants, allowing for effective monitoring of environmental pollutants. 

## 2. Synthesis and Fabrication of NPs

Currently, NPs are fabricated using mostly chemical and physical processes. Chemical techniques of synthesising NPs are more effective due to easy operation and control. Chemical techniques provide identical size and shape and possess the ability to design necessary functional groups on the surface that are required for use as nanosensor particles. In general, the inorganic NPs that are synthesised using solution-based chemical reactions, are capped by organic shells named stabilisers or surface-capping agents. These types of agents contributed to ensuring colloidal stability and potential surface modification. The agents prevent undesired aggregation and give the capability to attach a wide variety of functional groups and sites for biological modification. Nevertheless, producing monodispersed NPs with well-controlled particle size and shape is still a considerable challenge faced by nanotechnology research [74].

### 2.1. Gold Nanoparticles 

**Figure 1:**
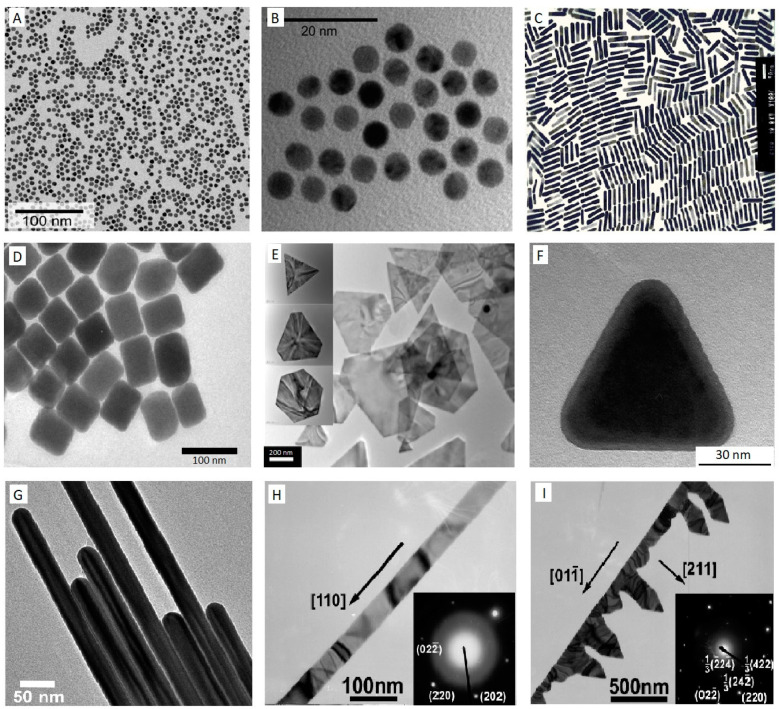
TEM images of Au nanostructures (**A**–**I**), Au nanospheres (**A**,**B**) [111], AuNRs (**C**) [100], Au nanocubes (**D**) [101], Au nanoplates (**E**) [104], Au nanoprism (**F**) [103], Au nanowire (**G**) [106], Au nanobelt (**H**), and Au nanocomb (**I**) [108]. Reprinted with permission from respective sources.

The intrinsic properties of AuNPs are governed by their shape, size, and structures. These remarkable characteristics have prompted a wide range of research to discover dependable and useful fabrication techniques for synthesising AuNPs with various structures, sizes, and shapes based on their application. In addition to the typical monodisperse colloidal spherical shape, different shapes of AuNPs have also been synthesised. A compilation of the more common shapes is shown in Figure 1. Various shapes can be obtained by employing different synthesis procedures as well as changing numerous parameters, including the condition of reactions, reactant concentration, and the nature of solvent [75,76]. The high chemical stability, unique size- and shape-dependent optical and electrochemical properties of AuNPs have made them a model NP in different fields of research, such as crystal growth [77,78,79], catalysis [80,81,82,83], nanosensors [84,85], electron-transfer mechanism [86,87], DNA/RNA assays [88,89,90], and self-assembly [74,91,92]. 

By far, the most popular and standard protocol used for obtaining monodisperse aqueous Au colloidal is a variation on the classic Turkevich citrate reduction route, called citrate–Au nanoparticles synthesis [74]. This method was first pioneered in 1951 [93] and later refined by G. Frens in 1973 [94] to control the dimensions of AuNPs by adjusting the ratio of the stabilising and reducing agents in Au suspensions. The principle of this technique lies in the reduction of Au^3+^ ions to Au^0^ atoms in the presence of reducing agents, for example, citrate [75]. Briefly, in this approach, a freshly prepared aqueous solution of sodium citrate tribasic dihydrate is added to a boiling solution of chloroauric acid (HAuCl_4_·H_2_O). During the first few minutes of the reaction, the colour of the solution changes from yellow to blue black to a deep wine red, suggesting the formation of AuNPs. In the next step, the sodium citrate initially functions as a reducing agent, which results in a reduction of Au^3+^ ions to neutral gold atoms. Furthermore, it acts as a stabilising agent and the negatively charged citrate ions stick to the AuNPs surface, providing the surface charge that repels the particles away from each other, thus preventing aggregation and precipitation. Typically, this method is applied to obtain modestly monodisperse spherical AuNPs suspended in water with the size of approximately 10–20 nm in diameter [95], by modifying the concentration of sodium citrate, various sizes of nanosphere can be achieved, mostly in a range of 16 nm–147 nm [75,96]. However, to produce smaller particle sizes or synthesise Au in organic solvents (oil–water interface), the Brust method was discovered in 1994 [97], in which AuNPs are developed in toluene with controlled diameters in the range from 1.5 to 5 nm. The method involves the transfer of an aqueous solution of Au ion to an organic solvent such as toluene using a phase transfer agent or surfactant-like tetraoctylammonium bromide (TOAB), followed by a reduction procedure with applying sodium borohydride (NaBH_4_) in the presence of an alkanethiol such as dodecanethiol. The organic phase of the solution represents a fast change in colour from orange to deep brown by the addition of NaBH_4_ [75,98]. Although spherical AuNPs with various sizes can be synthesised using Turkevich and Brust methods, AuNPs can also exist in various nanostructure forms such as rods [99,100], cubes [101,102], plate [103,104], prism [103,105], wire [106,107], belt [108,109], comb [108], etc. 

Monodispersed Au nanorods (AuNRs) with diverse aspect ratios are synthesised using a seed-mediated approach [99,110]. First, a seed solution needs to be prepared by mixing up an aqueous solution of cetyltrimethylammonium bromide (CTAB) and HAuCl_4_ before the quick addition of ice-cold NaBH_4_ under vigorous stirring which results in the formation of a brownish solution. Then, to grow nanorods (NRs), a growth solution with a suitable amount of CTAB, HAuCl_4_, AgNO_3__,_ and a weak reducing agent such as ascorbic acid (vitamin C) are mixed. The seed solution is then introduced to the colourless growth solution of the metal salt and left to age. A colour change based on the length of synthesised NRs can be observed in approximately 20 min. The length variation of the NRs is controlled by the amount of AgNO_3_ contained in the growth solution. Adding more AgNO_3_ leads to producing longer NRs [96,99]. The most widely preferred technique to alter the geometry of Au nanostructures in other shapes is to modify seed-mediated growth by changing seeds, reducing and structure-directing agents concentrations [75,76]. 

Niu et al. [101] report a modified seed-mediated growth methodology to change the shape and size of Au nanostructures to synthesis single-crystalline rhombic dodecahedral, octahedral, and cubic Au nanocrystals in a three-step procedure involving synthesising of AuNRs, overgrowing of the NRs into seeds, followed by using cetylpyridinium chloride as the surfactant. Huang et al. [103] discovered a route for controlling the size and shape of Au nanostructures by manipulating the concentration of CTAB while keeping the concentration of HAuCl_4_ unchanged without using any reducing agent. They successfully synthesised three-dimensional (3D) hexagonal and two-dimensional (2D) octahedral nanoplates and nanoprisms structure of Au nanocrystals. The amount of seed solution also plays a significant role in synthesising of AuNRs. Kim F et al. [106] reported synthesising Au nanowires by a three-step seeding method using significantly lower levels of seeds in the acidic growth solution. In another research, by reduction of HAuCl_4_ by ascorbic acid in aqueous mixed solutions of CTAB and the anionic surfactant sodium dodecyl sulfonate (SDS), Zhao N et al. [108] synthesised well-defined gold nanobelts along with unique gold nanocombs made of nanobelts. 

### 2.2. Carbon Allotropes

**Figure 2:**
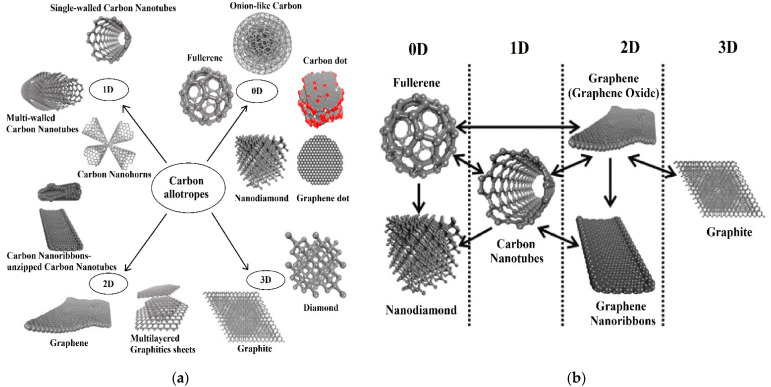
The structures of various allotropes of carbon (**a**), scheme which shows inter-conversion between different carbon nano-allotropes that emphasise the change in the dimension (**b**). The one-side arrow in section (**b**) reflects a transformation in one direction, while the two-sided arrow labels transformation in two directions. Reproduced with permission from [113]. Copyright © 2021 American Chemical Society.

Carbon and its allotropes have received significant attention in the field of sensing applications because of their unique exceptional properties, mainly in the form of a nanoscale [112]. The structures of different allotropes of carbon are shown in Figure 2 [113]. The carbon atom’s versatility sits in the variety of its chemical bonds which own wide sorts from sp^3^ to sp^2^, sp^1^, and combinations of them to yield amorphous or crystalline solids [112]. Diamond and graphite are the most popular crystalline forms of carbon. Diamond consists of carbon atoms in four-coordinated sp^3^, forming an extended 3D network, whose design is the chair conformation of cyclohexane. Graphite is made up of carbon atoms in three-coordinated sp^2^, forming planar sheets, whose motif is the flat six-membered benzene ring [114]. Lonsdaleite is a hexagonal crystallographic-structured carbon-based material that can be synthesised similar to a diamond at high static pressure and high temperature [115]. Fullerenes have attracted considerable attention by having a closed-cage structure consisting entirely of three-coordinate carbon atoms tiling the spherical or nearly spherical surfaces, which were accidentally discovered in 1985 by Kroto et al. [116,117] through exploring the nature of carbon represented in interstellar space [114]. Amorphous carbon is a non-crystalline solid carbon material lacking a long-range crystalline order. However, some short-range order is observed in the positions of the carbon atoms. Chemical bonds between atoms are different kinds of orbital configurations of sp^2^- and sp^3^-hybridised bonds with numerous concentrations of dangling bonds. The properties of amorphous carbon are significantly changed based on the formation methods and conditions [118]. GR is already known in nature as the most important component of graphite [119]. Geim and Novoselov were awarded the Nobel Prize in Physics in 2010 for discovering GR nanomaterials as a “wonder multifunctional material” [120]. GR has primarily been synthesised from graphite and carbon precursors using top-down [121] and bottom-up [122] methods, respectively. Epitaxial growth of GR on silicon carbide (SiC), and different metal substrates, such as Ni, Pt, Cu, Ir, Co, [123,124,125,126,127,128,129], solvothermal and organic synthesis [130,131,132,133] are some of the methods that have been identified for mechanical and chemical exfoliation of graphite. Chemical exfoliation of graphite can produce GO, which has been reported for over 150 years, with the first instance being carried out by B.C. Brodie in 1859. GO is insulating but easily dispersible in water because it has oxygenated functional groups attached to its basal plane and edges [134]. Via the reduction process, rGO can be generated from GO [135]. CNTs are the ordered, hollow GR-based nanomaterials made up of carbon sp^2^-hybridised atoms. They can be classified into the following 2 categories: (1) single-walled CNTs (SWCNTs), consisting of a single sheet of carbon that has been rotated into a tubular form, and (2) multi-walled CNTs (MWCNTs), which are comprised of several concentric SWCNTs having a mutual longitudinal axis [19,136]. Synthesis of CNTs has been conducted in various conditions. An active catalyst, a carbon source, and sufficient energy are needed for its synthesis [137]. Commonly used techniques for the production of CNTs include arc discharge, laser evaporation/ablation, chemical vapour deposition, electrolysis, sonochemical/hydrothermal, and solar technique [138]. The well-ordered pore structure and uniform pore size of GMCs make them attractive materials in various applications such as catalyst supports. They have a high surface area, significant graphite-like domains, enhanced conductivity, and efficient adsorption and desorption properties [139]. GMCs can be synthesised using different techniques, including catalytic graphitisation, high-temperature or/and high-pressure treatment of carbon precursors, and high-temperature chemical vapour deposition. Among these, the catalytic graphitisation method has received a lot of attention due to the lower temperature (900 °C) of thermal treatment, lowering the cost of graphitic carbon materials. The common GMCs synthesis steps consist of the preparation of a hard template, filling it with carbon precursors and catalysts, thermal treatment at high temperatures, and lastly, removing the template by dissolution or thermal treatment. Until now, catalysts such as Ni, Mn, Fe, and Co have been used for the hard templating catalytic graphitisation [140]. CNFs are one of the most important types of carbon fibres, and they’re a promising material for a variety of applications, including sensor electrode materials. Catalytic thermal chemical vapour deposition growth and electrospinning, followed by heat treatment are the two main methods used to produce CNFs [141]. CNHs are carbon nanomaterials (CNMs) with a conical shape made from a sp^2^ carbon sheet. In a variety of applications, they are a viable and practical substitute for CNTs and perhaps GR. Based on the approach utilised to inject energy into the carbon, synthesis processes could be divided into three groups, namely, arc discharge, laser ablation, and Joule heating. Unfortunately, as a result of aggregating into spherical clusters, their research and development has decreased. A new strategy of separating these “dahlia-like” clusters into individual CNH based on reduction with potassium naphthalenide has recently solved this constraint, and currently, they are produced in industrial quantities [142]. 

In the last two decades, CNMs have become one of the most exciting and extensively studied carbon materials, which attracted substantial attention in the electrochemical fields and found different applications due to their vast structural diversity and allotropic forms in many diverse areas. The classification of these materials depends on their dimensions. For example, zero-dimensional (0D) structures such as fullerenes, carbon dots, onion-like carbon, graphene dots, and nanodiamonds; one-dimensional (1D) form such as CNFs, CNHs, and CNTs (single and multi-walled); two-dimensional (2D) layered materials such as GR, graphene nanoribbons, and multi-layer graphite nanosheets; 3D structures such as the hybrid form consisting of multiple carbon nano-allotropes (GR-CNTs) [112,113]. Compared with other types of nanomaterials such as MNPs, transition metal dichalcogenides (TMDs), and metal oxide nanowires (NWs), CNMs have shown desirable aspects including high chemical stability, wide surface area to volume geometry, low cost, relatively inert electrochemistry, and rich surface chemistry for different types of redox reactions. Therefore, it has been used in a variety of sensors for highly sensitive and selective electrochemical determination applications, such as heavy metals, toxins, pesticides, etc. [112]. A new form of glass-like carbon (glassy carbon) has been introduced in electrochemistry for improved detection of targeted hazardous contaminants. It is an amorphous carbon allotrope produced by the controlled pyrolysis of an organic polymer, with a turbostratic structure in which poorly organised graphitic planes are arranged in ribbons as in polymers, giving rise to an isotropic material on average [143,144,145]. Krajewaska et al. [146] developed an electrochemical biosensor using glassy carbon electrodes (GCE) coated with SWCNTs and haemoglobin (Hb/SWCNT/GCE) for amperometric detection of acrylamide in water solutions. The existence of toxic acrylamide in a variety of foods, including potato crisps, French fries, and bread, was verified. The biosensor’s LOD was extremely low (1.0 × 10^−9^ M). The electrodes were found to be ideal for the sensitive detection of acrylamide in food samples following the verification test in a matrix obtained by water extraction of potato crisps.

## 3. Gold–Carbon Nanocomposites 

The significant privilege of using nanocomposites over conventional composites is to combine the attractive properties of various nanomaterials, which can greatly enhance the detection and degradation of hazardous environmental contaminants. It is critical to address the environmental protection and removal of pollution as some of the most serious global issues which need to be of concern as early as possible. Living in a healthy and clean environment is very important to human lives and well-being. Currently, the world is encountering a difficult challenge in meeting a growing requirement for clean, safe, and healthy environments. Lately, organic pollutants, toxic gases, pesticides, heavy metals, and other noxious chemicals in the air, soil, and water are the key factors that cause the surrounding environment to become worse. Even trace levels of contaminants can enter the human body and have acute effects on human health. Globally, the numbers of landfills and dumpsites, oil fields, private properties, military installations, industrial- and manufacturing-contaminated sites are staggering [21,147]. Nanotechnology, along with nanomaterials research, has the potential to discover reliable and powerful solutions for the determination and control of emerging contaminants in the surrounding environment [21]. 

**Figure 3:**
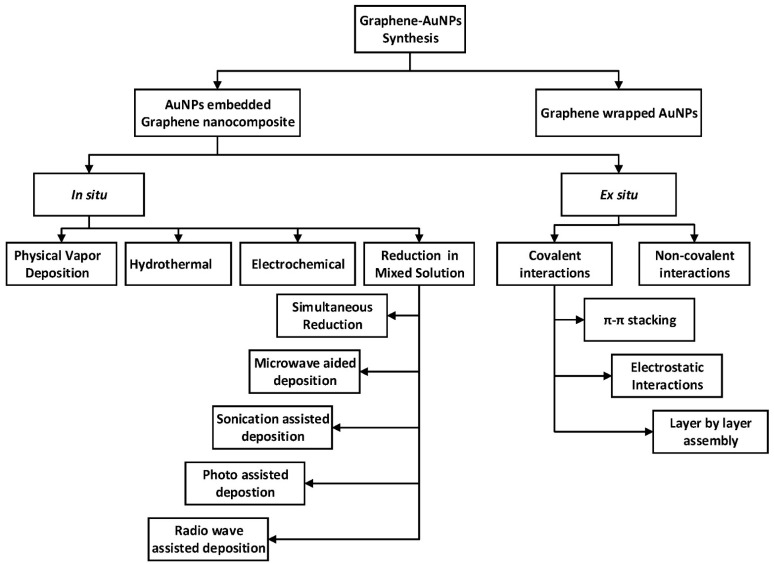
Schematic representation of the formation of graphene–AuNPs nanocomposites [161].

As previously mentioned, nanocomposites are a mixture of two or more phases that may contain various structures or compositional elements, in which the nanoscale system has at least one phase. Due to the small size of the structural unit and the high surface-to-volume ratio, these materials behave differently from previous composite materials. The amount of mixing between the two phases has a significant impact on the characteristics of composite materials [46,47]. A crucial challenge in the fabrication of nanocomposites is the capability to achieve the highest dispersion of nanoscale particles as well as maintaining this dispersion during the life cycle of the nanocomposite [148]. Increased dispersion of nanoparticles in the matrix of nanocomposites improves the achievement of a high load, resulting in a more uniform distribution [149]. The high loading of nanoparticles helps to obtain high-performance nanocomposites [150]. Recently, many applications based on gold–carbon nanocomposites have been developed, such as gold–graphene in biosensing [151], supercapacitor [152], bacteria detection [153], and dopamine sensing [154]; gold–carbon nanotube in drug delivery [155], biosensing [156], DNA detection [157], and solar cells [158]; gold–carbon nanofibers in energy storage [159], nanosensors [160], etc. In general, the methodology for the synthesis of gold–graphene hybrids can be classified into two main classifications, namely, gold-embedded graphene nanocomposites and graphene-wrapped gold nanoparticles [161]. As can be seen from Figure 3, gold-embedded graphene nanocomposite is synthesised using two different techniques—in situ and ex situ. Numerous synthesis processes fall within these two major headings, such as physical vapour deposition and electrostatic interaction methods. In the graphene-wrapped gold nanoparticles procedure, AuNPs of various sizes can be easily wrapped or encapsulated using GR, GO, or rGO because of their flexibility and 2D nature. Several ways of producing gold–carbon nanotube nanocomposites have been established, including direct and linked deposition of AuNPs on CNTs. In the direct deposition method, the nanostructures of gold are directly attached to CNTs without the use of any connecting molecules which can be classified into physical and wet chemical approaches. In linked deposition procedure, there are some linkages between CNTs and the gold nanostructures which can be classified as covalent or non-covalent. As direct deposition methods are mostly in situ, the AuNPs are less uniform due to different local variations on the CNTs. The high surface area of AuNPs and CNTs, together with their simple surface modification and great conductivity of CNTs, contribute to a wide range of gas sensing, biosensing, and electrochemistry applications [46].

Most of the environmental applications are focusing on the monitoring of gas, toxicant, and pesticide pollutants [162,163,164,165,166,167]. Therefore, this review is divided into these three sections. 

### 3.1. Gas Sensors

Gas sensors based on Au-functionalised carbon materials have been considered to obtain high response (ΔR/R0) because of their spillover effects (catalytic action of noble metal interface for gaseous dissociation and successive spreading of charged gaseous ions on anchoring substrate because of free electrons and high conductivity of these particles) at these nanoparticles [168]. The operation of most gas sensors is based on detecting a variation in the intrinsic electrical properties of the nanostructured material of the sensor in the presence of a test gas [18]. The sensing efficiency of gas sensors can be further enhanced via attachment with different MNPs such as Mg, Cr, Fe, Al, Co, Zn, Pd, Au, etc. [169,170,171,172,173,174]. Gas sensors play an important role in various industrial or domestic applications in diverse fields such as environmental monitoring, automotive industry, medical applications, military, and aerospace [175,176]. Although solid-state gas sensors have numerous advantages such as low cost and power consumption, small size, high sensitivity for detecting a wide range of gases in very low concentrations, they suffer from issues related to limited measurement accuracy and long-term stability [176]. Recent advances in nanotechnology and nanoscience provide remarkable opportunities to design the next generation of gas sensors by using novel nanostructures as sensing materials. The sensitivity of gas sensors is mostly determined based on parameters such as specific surface-to-volume ratio, which is much higher in sensors employing nanostructure rather than conventional microsensors. The nanostructured materials possess higher detection areas which leads them to have greater adsorption of gaseous species. As a result, their sensing capability is increased. This phenomenon makes them the best-sensing materials candidates for producing high-efficiency gas sensors [5].

**Figure 4:**
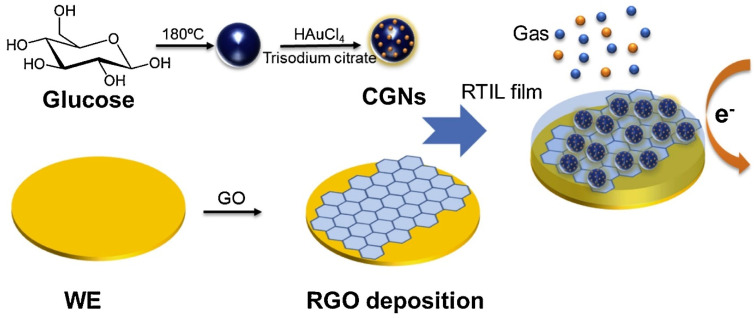
The modified rGO–CGN electrochemical gas sensor preparation procedures for oxygen sensing. Reproduced with permission from [171]. Copyright © 2021 Elsevier B.V. All rights reserved.

In 2019, Wan et al. [171] synthesised a novel and highly sensitive rGO-based electrochemical gas sensor with carbon–gold nanocomposites (CGNs) by glucose carbonisation and deposition of AuNPs via the hydrothermal procedure (Figure 4). This rGO–CGN customised gas sensor exhibited considerably improved existing reactions during oxygen sensing. The sensor was calibrated from 0.42% to 21% with good sensitivity, linearity, and reproducibility for oxygen detection.

A highly sensitive, flexible, and transparent gas sensor based on SWCNTs decorated with AuNPs in a facile and low-cost fabrication method was fabricated by Lee et al. [177]. Firstly, the films of SWCNT were spray-deposited on flexible and transparent PET substrates, then functionalised with AuNPs. The gas sensor reported acting as a low power enhanced sensitivity detector of NH_3_ up to 255 ppb (parts per billion) at room temperature in terms of electrical resistance variation of SWCNT films. This detection limit is one of the lowest values of concentration detected for nanotube-derived sensors. Du et al. developed a CNT/Au/SnO_2_ nanotubes hybrid via the in situ homogeneous depositions of Au and SnO_2_ nanocrystals onto the surface of CNTs through layer-by-layer (LbL) assembly technique [178]. This nanotube hybrid has been applied to develop highly sensitive gas sensors to detect CO gas at ambient temperature. In this method, by using the LbL assembly approach, first, a layer of polyelectrolyte type material, such as poly (diallyl dimethylammonium chloride) (PDDA) and sodium poly (styrene sulfonate) (PSS), was coated on the surface of CNTs to make it positively charged. Next, a mixture of an aqueous solution of HAuCl_4_ and trisodium citrate dihydrate was prepared to be added to the solution of polyelectrolyte modified CNTs. By mixing these two solutions, negatively charged AuCl_4_^−^ was adsorbed on the surface of positively charged modified-CNTs, and then the excess NaBH_4_ solution was gradually added to the prepared mixed solution, resulting in AuCl_4_^−^ reduction to Au and deposit on the surface of CNTs. Finally, a solution of SnCl_4_ was inserted into the mixture dropwise, which leads to the deposition of a layer of SnO_2_ onto the Au–CNTs hybrid (CNT/Au/SnO_2_). The synthesised gas sensor demonstrated a high level of sensitivity of nearly 70 and a superior response with less than 20 s recovery time for 2500 ppm concentration of CO at ambient temperature.

**Figure 5:**
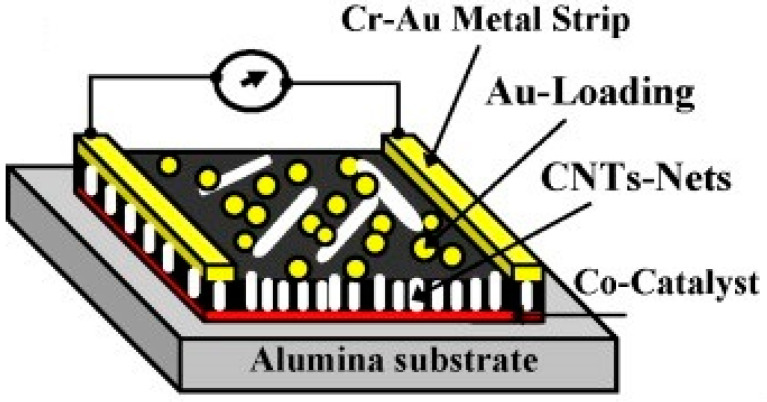
Schematic of the two-pole chemiresistor based on CNTs networks, grown by radiofrequency plasma-enhanced chemical vapour deposition onto alumina substrate coated with cobalt (Co) catalysts, surface-functionalised with Au nanoclusters. Reproduced with permission from [180]. Copyright © 2021 Elsevier B.V. All rights reserved.

A hybrid metal decorated MWCNTs thin film and WO_3_ nanopowders were functionalised to develop a gas sensor for the detection of NO_2_ gas [179]. The 50–100 nm WO_3_ nanopowders with a hexagonal structure (hex–WO_3_) were prepared via acidic precipitation from the solution of sodium tungstate. In this novel structure, MWCNTs were inserted into the medium of hex–WO_3_ at a very low concentration. Metallic nanoclusters of Au and Ag were used as catalysts to improve the sensing performance of the gas sensors. They reported that the use of the MWCNTs film lowered the current working temperature (150–250 ℃) of the sensor. Therefore, the hex–WO_3_/MWCNTs mixtures were responsive to hazardous NO_2_ gas at room temperature. Their fabricated hybrid material mixtures were capable of detecting as low as 100 ppb of NO_2_, without heating the sensor substrates during operation. Penza et al. [180] have exhibited the impact of CNT–Au nanoclusters hybrid structure on gas sensing performance of a chemiresistor, at varying working temperatures (25–250 ℃), (Figure 5). When exposed to an oxidising NO_2_ gas, CNTs and Au-modified CNTs demonstrate a p-type response with a reduction in electrical resistance and an escalation in resistance when exposed to reducing gases such as NH_3_, CO, N_2_O, H_2_S, and SO_2_. Upon deposition of Au nanoclusters on the CNT network, enhanced gas response (NO_2_, H_2_S, and NH_3_) up to a low limit of the sub-ppm level was exhibited. They also reported good repeatability of the electrical response, up to 200 ppb of NO_2_ gas at 200 ℃.

**Figure 6:**
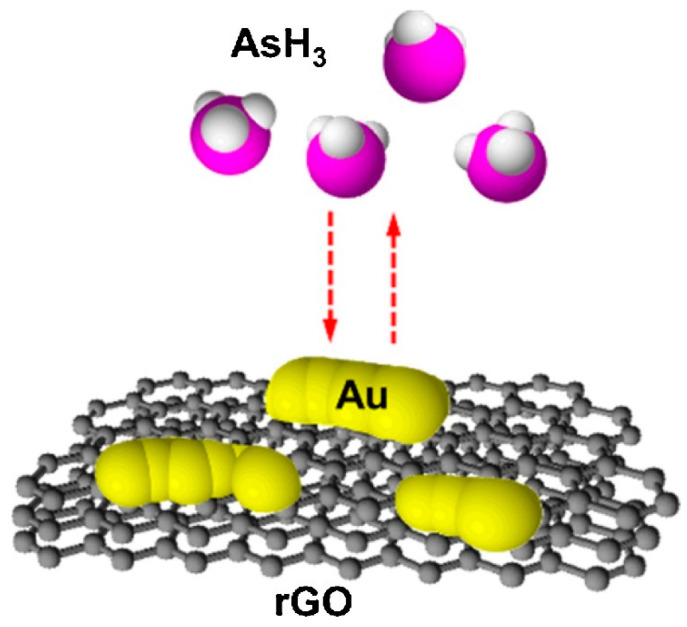
The graphic of the AsH_3_ gas sensor based on Au-modified rGO. Reproduced with permission from [163]. Copyright © 2021 Elsevier B.V. All rights reserved.

Cittadini et al. [15] reported the production of the optical gas sensor using GO coupled with AuNPs. Firstly, a monolayer of AuNPs was prepared and chemically attached to the functionalised, fused SiO_2_ substrate, followed by spin-coating deposition of GO flakes on AuNPs. They investigated gas-sensing performances of the sensor upon exposure to reducing and oxidant gases such as H_2_, CO, and NO_2_ with (10,000 ppm and 100 ppm), (10,000 ppm), and (1 ppm) concentration, respectively. In particular, the surface plasmon resonance (SPR) band shifted towards exposure to these gases. The SPR response from the sensor can be explained due to the strong interaction of the Au–GO hybrid via the electron transfer activity of the AuNPs and the two-dimensional sheet of sp^2^-hybridised GO carbons. A highly sensitive AsH_3_ gas sensor was fabricated using a thin layer of Au and rGO nanosheets on an interdigitated array electrode (IDE), (Figure 6) [163]. The conductivity Au/rGO sensor was observed by the continuous generation of AsH_3,_ created by chemical reduction of aqueous arsenite with borohydride in an acidic medium and vaporisation of the hydride to test the sensor. They tested the response of Au, rGO, and Au/rGO IDE sensors to AsH_3_ vapour. The Au/rGO sensor resistance was decreased when it was exposed to AsH_3_. However, gas sensors assembled with only Au or rGO did not show sensitivity to AsH_3_. The rise in conductivity of the gas sensor possibly appeared due to the AsH_3_ depleted adsorbed oxygen on the Au islands and therefore resulted in the increase of hole conduction in the rGO film. By optimisation of the volume of Au and rGO, reduction of rGO, and working temperature, a LOD of 0.01 ppmv for this gas sensor was obtained. Interference responses of the sensor to other gases and vapours were also examined. Young et al. [181] reported the production of ethanol gas sensors by the growth of high-density CNTs on oxidised Si substrate, with and without adsorption of AuNPs on the surface of nanotubes. It was reported that the incorporation of AuNPs could significantly enhance the sensitivity of the device. A large (3.28%) sensitivity with adsorption of Au (when the concentration of the injected ethanol gas was 800 ppm) at room temperature was achieved. Moreover, the response stability and speed of the synthesised sensor were both good.

### 3.2. Toxicant Sensors

The widespread production and use of synthetic and natural chemicals by society have led to the release of huge amounts of toxic materials into the environment. Thus, their persistence has necessitated the development of fast and cost-effective toxicity tests to protect humans and other organisms [182]. Toxicity assays are necessary for the avoidance of environmental destruction and obstacles to the health of the human body. Toxicity testing has been extensively required in the field of environmental protection as well as in the diagnosis and food fields. Toxicant materials are usually detected by employing chemical or physical procedures, such as HPLC and ion-selective electrodes (ISEs). Nevertheless, the toxicity of such chemical materials cannot be measured [183]. Highly sensitive detection and determination of toxic substances are of great importance for people’s health and environmental protection [46].

**Figure 7:**
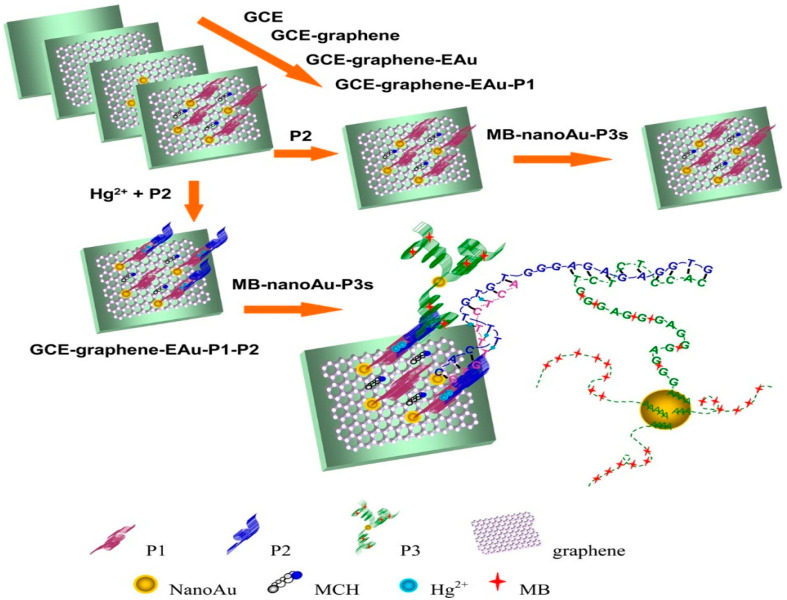
The outline diagram of preparation procedures of the MPH/SP@AuNPs/MWCNTs/GCE toxicant sensor for the detection of mercury. Reprinted with permission from [184]. Copyright © 2021, American Chemical Society.

In 2015, Zhang et al. [184] established a sensor for attomolar detection (0.001 aM) of mercuric ions (Hg^2+^) by electrodepositing of GR and nanoAu (GR-EAu) on a surface of GCE (GCE-GR-EAu). Figure 7 represents the schematic approach of the electrochemical sensor platform for the sensitive detection of Hg^2+^. Three ss-DNA probes, namely, a 10-mer thymine-rich DNA probe (P1), a 22-mer thymine-rich DNA probe (P2), and a 29-mer guanine-rich DNA probe (P3) were designed for sensitive and selective detection of the target. It is recognised that DNA can cooperate with a variety of metal ions such as Hg^2+^. In this work, the presence of Hg^2+^ is caused by the metal-mediated DNA duplexes between P1 and P2 due to thymine–Hg^2+^–thymine (T–Hg^2+^–T) coordination chemistry. NanoAu carriers functionalised with DNA-labelled methyl blue (P3) were used as a signal amplification to enable such a low limit of detection.

**Figure 8:**
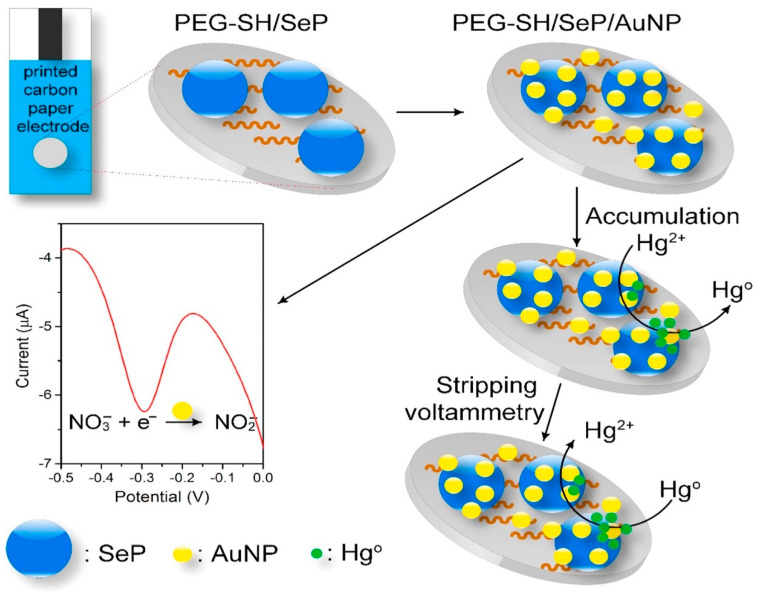
The graphic design of the fabrication procedure and reactions occurring on the SePs/AuNPs modified disposable carbon paper electrode for the detection of NO_3_^−^ and Hg^2+^. Reprinted with permission from [185]. Copyright © 2021 Elsevier B.V. All rights reserved.

Figure 8 shows the schematic illustration of the modification process of a simple disposable dual electrochemical sensor for the detection of nitrate (NO_3_^–^) and Hg^2+^ based on the deposition of selenium particles (SePs) and AuNPs onto the surface of a carbon-printed paper, having PEG–SH as a linker. In this sensor, SePs acts as an absorbing agent for Hg^2+^ because of the high binding affinity to mercury that can improve the anodic stripping voltammetry of mercury and the AuNPs catalyse the reduction of NO_3_^–^ and Hg^2+^. The PEG–SH/SePs/AuNPs sensor exhibited enhanced sensitivity to detect NO_3_^–^ and Hg^2+^ with LOD of 8.6 mM and 1.0 ppb, respectively [185].

Compton et al. [186] reported a fast and efficient electrochemical detection of arsenic (III) in aqueous media by electroless deposition of AuNPs on MWCNTs through in situ reductions of HAuCl_4_ by NaBH_4_. The modified MWCNTs were immobilised on a GCE surface through the evaporation of solutions in chloroform. With the modified electrode in As (III) solutions, anodic stripping voltammetry was performed. The complete process from modification of electrode to the detection of arsenic has been achieved in just a few minutes. A high sensitivity (1985 µA M^−1^ with square wave voltammetry) and a very low LOD (0.1 g L^−1^) were routinely obtained. The Au-modified MWCNTs exhibit a long lifetime and generate accurate measurements over 10 months. An electrochemical disposable sensor for highly sensitive detection of bisphenol A (BPA) in an aqueous solution was fabricated via an easy, low-cost, and environmentally friendly approach [187]. The fabrication of the sensor was performed through the synthesis of rGO/CNT/AuNPs nanocomposites on the screen-printed electrode (SPE) at room temperature. The prepared sensor demonstrated a vast working range, great selectivity, and sensitivity over BPA in a manner that electrodeposition of AuNPs considerably improved the electron transfer and electrocatalytic capabilities over BPA. They reported that, in an optimised condition, differential pulse voltammetry (DPV) showed linear existing responses for BPA concentrations of 1.45 to 20 and 20 to 1490 nM, with a determined ultralow LOD of 800 pM.

Kan et al. [188] established the fabrication of a flexible disposable graphite-based electrochemical sensor for individual and sensitive simultaneous detection of catechol (CC) and hydroquinone (HQ). The fabrication of the sensor was performed through the electrodeposition of AuNPs onto an exfoliated graphite paper (EGP) as a support electrode to fabricate the desired (AuNPs/EGP) sensor. The AuNPs/EGP sensor exhibited a wide linear range (5.0 × 10^−7^ to 1.0 × 10^−4^ mol/L and 7.0 × 10^−8^ to 1.0 × 10^−4^ mol/L) for CC and HQ detection, as well as LOD (S/N = 3) of 4.13 × 10^−8^ mol/L and 2.73 × 10^−8^ mol/L, respectively. Zhu et al. [189] constructed a unique and effective electrochemical sensor based on AuNP-decorated, rGO-modified GCE via one single step for detection of trace level of iron by DPV. In their study, the modified electrode was fabricated with 5-Br-PADAP (2-(5-Bromo-2-pyridylazo)-5-diethylaminophenol) as complex agents for the sensitive determination of Fe^3+^ in real coastal water samples. rGO served as a support to provide a large specific surface area for AuNPs. As a result, the electrochemical reduction of Fe(III)-5-Br-PADAP was induced. The low LOD of 3.5 nM with a linear response in a range of 30 nM to 3 µM was achieved.

### 3.3. Pesticide Sensors

The term pesticide is often used for a wide range of chemicals that successfully apply to eliminate and/or control a variety of animals or plant pests and diseases. Pesticides according to their purpose of use can be categorised as insecticides, herbicides, fungicides, or a range of other kinds. Numerous chemical compounds such as arsenic, organophosphates, pyrethroids, carbamates, and nitrophenol derivatives are involved as pesticides. Pesticides can be classified in several ways, for example, chemical structure, biological target, and safety profile. Due to the high level of toxicity, environmental agencies have established a top admissible rate for their contaminant levels in surface and drinking water. Based on their water solubility, they either settle in the soil or permeate the surface waters and groundwater. Pesticide residues and their degradation products can stay in vegetables, living organisms, and water sources, and their concentration will increase as they climb the food chain. Due to their toxic effects, even at trace levels, there is an increasing interest to develop effective systems for sensing, monitoring, breaking down, and/or removing them. The increasing research interest in this area has generated countless attempts to produce systems to detect and degrade pesticides by applying different types of nanomaterials, such as MNPs, CNTs, GR, magnetic nanoparticles, and/or quantum dots [190].

**Figure 9:**
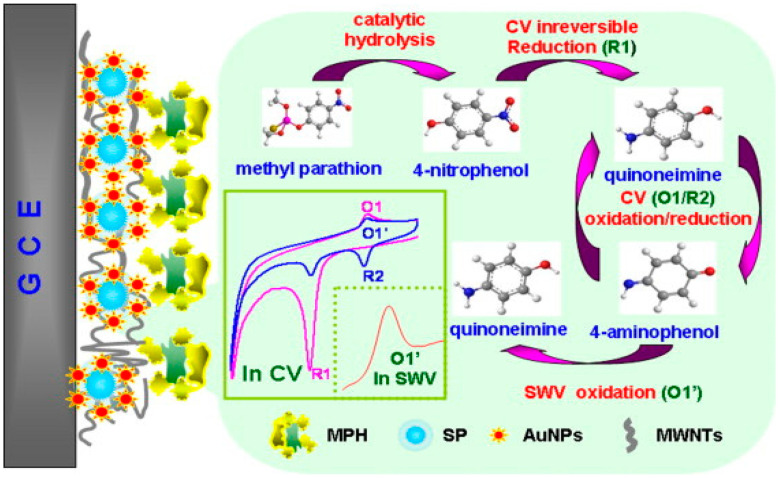
MPH biosensor preparation procedures for methyl parathion determination using modified SP@AuNPs/MWCNTs electrode. Reprinted with permission from [191]. Copyright © 2021 Elsevier B.V. All rights reserved.

Jha and Ramaprabhu [167] fabricated a disposable and sensitive biosensor by modifying a GCE with AuNPs and MWCNTs for the detection of paraoxon. AuNPs spread onto the surface of MWCNTs to form an Au–MWNT hybrid exploiting high electron transfer rate and significant immobilisation sites for bioenzymes, which merge with the high electrocatalytic activity of MWNTs towards thiocholine electrooxidation at the low potential in this nanocomposite. Au–MWNTs allow the detection of paraoxon at low potential amperometric levels without the use of a redox mediator. Very high sensitivity up to a concentration of 0.1 nM for the model analyte paraoxon has been achieved. This nanocomposite-based biosensor could as well be used for the detection of other organophosphorus (OP) combinations. In 2010, Du et al. [166] described the fabrication of amperometric acetylcholinesterase (AChE) biosensor, based on an Au–MWCNT–Chitosan-modified electrode, with LOD of 0.6 ng mL^−1^ for malathion. The synthesised biosensor demonstrated suitable fabrication reproducibility, appropriate stability, rapid response, and low LOD, therefore offering a novel promising device with possible application in biomonitoring of OPs exposure and detection of more toxic mixtures against AChE. A highly sensitive and specific nanocomposite biosensor for the detection of methyl parathion was developed via the formation of AuNPs on silica particles (SP@AuNP) mixing with MWCNTs (SP@AuNPs/MWCNTs) on the surface of a GCE and further covalent immobilisation of methyl parathion hydrolase (MPH) (Figure 9) [191]. The square wave voltametric (SWV) responses displayed a LOD of 0.3 ng mL^−1^, along with a linear response to the concentrations of methyl parathion in the range from 0.001 µg mL^−1^ to 5.0 µg mL^−1^. In garlic samples, the recovery test with known quantities of methyl parathion resulted from 95.0% to 102.3%, showing the strong precision of this biosensor.

**Figure 10:**
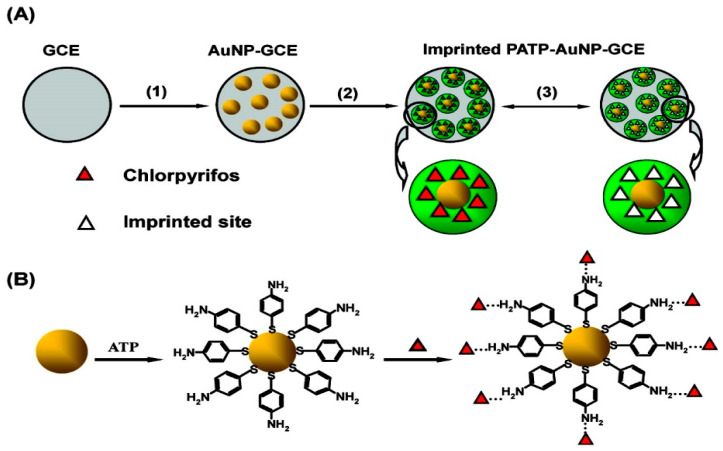
(**A**) Working principle of the imprinted PATP–AuNP–GCE: (1) AuNPs are electrodeposited on the surface of the GCE; (2) ATP electropolymerisation on the AuNP–GCE surface; (3) CPF removal/rebinding on the imprinted sites of the imprinted PATP–AuNP–GCE. (**B**) The graphic map for the adsorption of the ATP molecule on the surface of AuNP, followed by self-assembly of CPF at the ATP-modified AuNP–GCE. Reprinted with permission from [193]. Copyright © 2021, American Chemical Society.

Liu et al. [192] developed a novel AChE biosensor based on 3-carboxyphenylboronic acid (CPBA)/rGO–AuNP-nanocomposite-modified electrode for high sensitivity amperometric detection of organophosphorus (chlorpyrifos and malathion) and carbamate (carbofuran and isoprocarb) pesticides. The biosensor represents excellent sensitivity due to the outstanding properties of AuNPs and rGO, which promote electron transfer reactions and enhance the electrochemical response. The LOD of 0.1, 0.5, 0.05, and 0.5 ppb for chlorpyrifos, malathion, carbofuran, and isoprocarb, were achieved, respectively. In Figure 10, Xie et al. [193] showed a sensor protocol based on the molecularly imprinted polymer (MIP), that is synthesised in situ at an electropolymerised polyaminothiophenol (PATP) membranes on the surface of AuNP-modified GCE for the electrochemical detection of chlorpyrifos (CPF). A high ratio of imprinted sites is created by combining surface molecular self-assembly with electropolymerised molecular imprinting on the greater surface area of an AuNP-modified electrode, thus providing an ultrasensitive electrochemical detection of an organophosphate pesticide. A LOD of 0.33 μM for CPF along with a linear relationship in the range from 0.5 to 10 μM CPF was obtained.

A nanocomposites hybrid biosensor consists of AuNPs and chemically reduced graphene oxide nanosheets (cr-Gs) (AuNPs/cr-Gs) for the detection of organophosphate pesticides was developed via in situ deposition of AuNPs and AChE on cr-Gs (AChE/AuNPs/cr-Gs) in the presence of PDDA. In this study, PDDA acts as a stabiliser for cholinesterase with high activity and loading efficiency and a dispersible medium for AuNPs. The ultrasensitive LOD of 0.1 pM for paraoxon was obtained [194].

To compare the ability of different nanocomposites designs, we described the comparison of reviewed sensors/biosensors based on various substrates and strategies for environmental contaminant sensing in Table 1.

## 4. Conclusions and Future Perspectives

Environmental damage, due to the rapid growth of the world population and increasing globalisation, is a serious cause for concern. Over the past few decades, the development of novel treatment techniques for the detection, determination, and monitoring of hazardous environmental contaminants by high-level of accuracy, precision, reproducibility, and low LODs have shown extremely interesting tasks for the scientific society. The emerging nanoscience and nanotechnology techniques have provided insight to design novel nanocomposite materials with unique properties and structures to achieve cooperatively enhanced performance for pollutant elimination. The attractive chemical and physical properties of gold and carbon nanomaterials make them remarkably fascinating for use in labels or transducing systems for electrochemical or optical sensors and biosensors. AuNPs have been the desired nanoparticles for nearly all nanomaterial-based optical detection techniques. Similar to AuNPs, carbon nanomaterials are also the most widely used materials in electrochemical transducing systems. Their considerable properties, such as large specific surface area, excellent electrical conductivity, high surface free energy, high chemical, and thermal stability, and strong mechanical strength make them an ideal candidate to be used as a support in Au nanocomposites matrix to fabricate various electrochemical sensor/biosensor for environmental applications. It is expected that hybrids of AuNMs and CBNs should always be the most important focus of this research direction because they offer quite promising results due to the high sensitivity and selectivity of such combinations for the analysis of environmental contaminants. Nevertheless, we need to know that these achievements (e.g., a low and ultrasensitive LOD) are still in the phase of laboratory testing, and much research is needed to take these achievements to an industrially applicable and commercially viable device. A better understanding of the various shapes of AuNPs and their possible interaction with other nanomaterials, especially carbon nanomaterials, is necessary. In addition, future efforts should concentrate on developing better detection mechanisms using different disciplines and technologies to propose improved hybrid structures using AuNPs and carbon nanomaterials.

## Figures and Tables

**Table 1:** Sensitivity/LOD comparison of the reviewed nanocomposites sensing platform.

Sensors	Nanocomposites	Modification	Detected Items	Sensitivity/LOD	Ref
Gas sensor	rGO–CGN	Carbon–gold nanocomposites (CGN) on an rGO-based electrochemical gas sensor.	O_2_	Sensitivity of 0.289–0.168 μA/% O_2_ for low and high concentration range, respectively.	[171]
	SWCNT–AuNPs	SWCNT films spray deposited on transparent and flexible plastic substrates and then decorated with AuNPs.	NH_3_	255 ppb	[177]
	CNT/Au/SnO_2_ nanotubes	CNT/Au/SnO_2_ nanotubes synthesised via homogeneous coating of Au and SnO_2_ nanocrystals on CNTs.	CO	Sensitivity of about 70 Ig/Ia for 2500 ppm concentration of CO.	[178]
	Au-MWCNTs/hex–WO_3_	Metal decorated MWCNTs embedded into the hex–WO_3_ nanocomposites.	NO_2_	100 ppb	[179]
	Au-modified CNTs networks	Au nanoclusters deposited onto CNTs networks by sputtering.	NH_3_, CO, N_2_O, H_2_S, SO_2_	200 ppb NO_2_	[180]
	AuH–rGO	GO flakes deposited over a monolayer of AuNPs, chemically attached to a functionalised, fused silica substrate.	H_2_, CO, NO_2_	Sensitivity of 0.1% (for 100 ppm) up to 0.5% (for 10,000 ppm) for H_2_ and a variation of 0.1% for 1 ppm NO_2_, while CO not detected.	[15]
	Au/rGO	rGO-modified with a thin gold film on an interdigitated array electrode.	AsH_3_	0.01 ppmv	[163]
	Au–CNT	CNTs from a SiO_2_/Si substrate transferred to the flexible substrate and deposited with a controlled load of Au.	Ethanol Gas	Sensitivity of 5.39% for 800 ppm concentration of ethanol gas.	[181]
Toxicant sensor	GCE–GR–EAu	GR and nanoAu electrodeposited on the surface of GCE, then functionalised with the 10-mer thymine-rich DNA probe.	Hg^2+^	0.001 aM	[184]
	PEG–SH/SePs/AuNPs	Disposable carbon paper electrodes functionalised with SePs and AuNPs.	NO_3_^–^, Hg^2+^	8.6 µM and 1.0 ppb for NO_3_^–^ and Hg^2+^.	[185]
	Au-MWCNTs	AuNPs deposited on MWCNTs via reduction of HAuCl_4_ by NaBH_4_ followed by fixing it onto the GCE surface via evaporation of a suspension in chloroform.	As(III)	Sensitivity of 1985 μA/μM with square wave voltammetry and a LOD of 0.1 μg/L.	[186]
	RGO/CNT/AuNPs	GO/CNT nanocomposite reduced to RGO/CNT on SPE, followed by electrochemical deposition of AuNPs on modified SPE.	BPA	800 pM	[187]
	AuNPs/EGP	AuNPs electrodeposited on EGP to fabricate AuNPs/EGP sensor.	CC, HQ	4.13 × 10^−8^ mol/L and 2.73 × 10^−8^ mol/L for CC and HQ.	[188]
	GCE/rGO/AuNPs	A modified GCE based on the rGO and AuNPs fabricated with 2-(5-bromo-2-pyridylazo)-5-diethylaminophenol (5-Br-PADAP) as complexing agents.	Fe(III)	3.5 nM	[189]
Pesticide sensor	Au–MWNTs–GCE	AuNPs dispersed on the outer surface of MWNTs used to modify GCE.	Paraoxon	0.025 ppb	[167]
	AuNP–CHIT/GCE, MWCNT–Au–CHIT/GCE	Chitosan modified GCE (CHIT/GCE) coated with AuNPs and MWCNT–Au nanocomposites to fabricate AuNPs modified GCE (AuNP–CHIT/GCE) and MWCNT–Au nanocomposites modified GCE (MWCNT–Au–CHIT/GCE), respectively.	Malathion	0.6 ng/mL	[166]
	MPH/SP@AuNPs/MWCNTs/GCE	The sensing film prepared via the formation of AuNPs on SP (SP@AuNP), mixing with MWCNTs on the surface of a GCE followed by covalent immobilisation of MPH.	Methyl parathion	0.3 ng/mL	[191]
	CPBA/AuNPs/RGO-CS/GCE	An amperometric biosensor based on immobilising acetylcholinesterase on the modified GCE with nanocomposites of CPBA/rGO–AuNPs.	Chlorpyrifos, malathion, carbofuran, isoprocarb	0.1, 0.5, 0.05, and 0.5 ppb for chlorpyrifos, malathion, carbofuran, and isoprocarb, respectively.	[192]
	PATP–AuNP–GCE	Electropolmerisable PATP assembled on the AuNPs at the surface of GCE by the formation of Au-S bonds, then, the CPF template assembled onto the monolayer of ATP through the hydrogen-bonding interaction between amino group and CPF.	Chlorpyrifos	0.33 μM	[193]
	AuNPs/cr-Gs	In the presence of PDDA, a nanohybrid of AuNPs and cr-Gs synthesised by the growth of AuNPs on the surface of graphene nanosheets. Then, an enzyme nanoassembly (AChE/AuNPs/cr-Gs) was prepared by self-assembling of AChE on AuNP/cr-Gs nanohybrid.	Paraoxon	0.1 pM	[194]
